# Metabolic reprogramming networks in the gastric cancer tumor microenvironment: an integrated axis of nutrient competition, metabolic crosstalk, and immunosuppression

**DOI:** 10.3389/fimmu.2026.1801318

**Published:** 2026-07-09

**Authors:** Xiaona He, Jie Liu, Wenjie Wang

**Affiliations:** 1 Department of Critical Care Medicine, The Second Hospital & Clinical Medical School, Lanzhou University, Lanzhou, China; 2 Department of Tumor Surgery, The Second Hospital & Clinical Medical School, Lanzhou University, Lanzhou, China; 3 Department of General Surgery, The Second Hospital & Clinical Medical School, Lanzhou University, Lanzhou, China

**Keywords:** gastric cancer, immune suppression, metabolic crosstalk, metabolic reprogramming, nutrient competition, tumor microenvironment

## Abstract

Gastric cancer ranks among the most prevalent malignant tumors globally, with its immunotherapeutic efficacy constrained by the intricate tumor microenvironment (TME). This review provides a systematic elucidation of the dynamic interaction axis of “nutrient competition-metabolic crosstalk-immunosuppression” within the TME of gastric cancer, approached from a network perspective. Initially, the hypoxic, acidic, and nutrient-deficient conditions of the TME establish a metabolic pressure foundation, compelling cancer and immune cells to engage in intense competition for essential nutrients, thereby inducing a state of metabolic deprivation in effector T cells. Subsequently, this competitive dynamic results in the accumulation of immunosuppressive metabolites, including lactic acid, adenosine, kynurenine, and prostaglandin E2. These metabolites, rather than being mere waste products, form a complex metabolic crosstalk network that actively suppresses effective immune function and reshapes the immunosuppressive niche through mechanisms such as receptor signaling and epigenetic modification. Ultimately, this multi-layered metabolic reprogramming network collectively facilitates immune evasion and resistance to immunotherapy in gastric cancer. A comprehensive analysis of the network is anticipated to furnish a vital theoretical foundation for the development of novel therapeutic strategies. These strategies may include targeting pivotal metabolic nodes, alleviating immunosuppression, and integrating immune checkpoint blockade, all of which are expected to enhance the clinical prognosis of patients with gastric cancer.

## 1 Introduction

Gastric cancer is one of the most prevalent malignant tumors of the digestive tract globally, with its incidence and mortality rates ranking fifth among all cancers (1). Currently, radical surgery combined with perioperative chemotherapy/radiotherapy remains the cornerstone for resectable gastric cancer. Nevertheless, for those with locally advanced unresectable or metastatic gastric cancer, therapeutic advancements remain constrained. In recent years, immune checkpoint inhibitors (ICIs), particularly those targeting PD-1/PD-L1, have catalyzed the evolution of systemic treatment strategies for gastric cancer. Notably, the CheckMate 649 trial has demonstrated the survival benefits of combining Nivolumab with chemotherapy as a first-line treatment for advanced gastric, gastroesophageal junction, and esophageal adenocarcinomas (2). Similarly, the KEYNOTE-859 study indicates that pembrolizumab combined with chemotherapy confers an overall survival benefit while maintaining a manageable safety profile (3). Nevertheless, the sustained clinical response predominantly targets specific beneficiary subgroups, while primary or secondary drug resistance remains prevalent. This indicates that the tumor microenvironment (TME) plays a crucial role in influencing the sensitivity to and the evolution of drug resistance in immunotherapy, alongside the genomic variations of tumor cells (4).

In the TME of gastric cancer, the presence of abnormal vasculature and the heightened metabolic demands of cancer cells result in a pervasive state of metabolic stress, characterized by conditions such as hypoxia, acidosis, and a deficiency of essential nutrients, including glucose and amino acids (5, 6). Furthermore, the metabolic activities of cancer cells, along with certain immunosuppressive cells, generate a significant quantity of immunosuppressive metabolites, including lactic acid, adenosine, and reactive oxygen species (ROS) (7–9). These metabolites function not merely as metabolic byproducts but also as potent signaling molecules, forming a complex network of metabolic crosstalk. Through various mechanisms within the TME, these molecules inhibit effector immune functions and/or facilitate the expansion of cell subsets with immunosuppressive capabilities (10–12).

Consequently, a systematic analysis of this axis, characterized by the interplay of nutrient competition, metabolic crosstalk, and immune escape, is essential for elucidating the fundamental nature of the immunosuppressive microenvironment in gastric cancer and for identifying novel intervention targets. The innovation of this review resides in its comprehensive integration of diverse metabolic phenotypes and immunological outcomes in gastric cancer through a network perspective. This approach transcends the traditional focus on isolated metabolic pathways or immune cell functions, instead highlighting the dynamic interactions and synergistic relationships among nutrient competition, metabolic crosstalk, and immunosuppression. This review comprehensively examines metabolic reprogramming in the gastric cancer TME, focusing on metabolic stress, intercellular nutrient competition, key nodes within the metabolic crosstalk network, and the mechanisms by which metabolism drives immune evasion. By integrating these aspects, it aims to provide a theoretical framework for developing effective metabolism-targeting immunotherapies and to offer a scientific reference for improving patient prognosis.

## 2 The metabolic landscape of the immunosuppressive microenvironment in gastric cancer

The gastric cancer TME constitutes a complex metabolic niche characterized by chronic hypoxia, acidosis, and localized depletion of essential nutrients. These features are not isolated phenomena; rather, they are interrelated and synergistic, collectively creating an environment that is highly inhospitable to infiltrating immune cells, particularly those with anti-tumor effector functions. Conversely, this environment is more favorable to the survival of immunosuppressive cells.

Hypoxia is a prevalent characteristic of solid tumors. Under hypoxic conditions, tumor cells can enhance the transcriptional processes related to glucose transport and the glycolytic pathway by stabilizing hypoxia-inducible factor 1-alpha (HIF-1α), a key transcriptional regulator. This stabilization promotes a preferential shift towards glycolysis for lactic acid production, even in the presence of adequate oxygen levels (13, 14). Acidosis arises as a direct consequence of a metabolic profile predominantly governed by glycolysis. Lactic acid, rather than merely being a metabolic waste, functions as a signaling molecule and metabolic substrate that can influence the fate of immune cells and contribute to the maintenance of ecological homeostasis in immunosuppression (15, 16). Nutrient scarcity within the TME arises from the interplay between excessive cellular consumption and inadequate vascular perfusion. Tumor cells and CAFs exhibit elevated glucose uptake, thereby depleting the glycolytic substrates essential for effector T cells, which consequently leads to energy deprivation and functional exhaustion.

Hypoxia, acidosis, and nutritional deficiency should not be viewed as isolated pathophysiological phenomena; rather, they represent a three-pronged of metabolic stressors that form the foundational basis for immune suppression within the TME of gastric cancer. These factors collectively constrain the anti-tumor immune response through three primary dimensions: energy supply, environmental pH, and nutritional deficiency. This triad establishes conditions conducive to subsequent metabolic crosstalk and immune evasion. Furthermore, the presence of hypoxia, acidosis, and nutritional deficiency within the gastric cancer TME collaboratively facilitates the accumulation of various immunosuppressive metabolites. **Table 1** delineates the origins, mechanisms, and immunological impacts of these critical metabolites.

**Table 1:** Key immunosuppressive metabolites in the gastric cancer TME: sources, mechanisms, and impacts.

Metabolite	Primary source	Key molecular mechanisms	Impact on immune cells	References
Lactate	Glycolysis in tumor cells (Warburg effect)	Activates GPR81 signaling; Induces histone lactylation; Triggers SREBP2-mediated lipid signals	Inhibits cytotoxicity of CD8^+^ T and NK cells; Promotes Treg recruitment; Drives M2 macrophage polarization	(25, 33–36)
Adenosine	Hydrolysis of extracellular ATP via CD39/CD73	Activates A2A receptors; Elevates intracellular cAMP levels; Inhibits TCR signaling	Suppresses T cell proliferation and cytokine production; Enhances Treg suppressive function	(37–39)
Kynurenine	Tryptophan catabolism mediated by IDO1/TDO	Acts as a ligand for the aryl hydrocarbon receptor (AHR)	Induces T cell exhaustion; Promotes Treg differentiation; Facilitates immune tolerance	(40–43)
Prostaglandin E2 (PGE2)	Arachidonic acid metabolism catalyzed by COX-2	Binds to EP2/EP4 receptors; Activates PKA/CREB pathways	Impairs Dendritic Cell (DC) maturation; Promotes MDSC expansion; Upregulates PD-L1 expression	(44–47)
Nitric Oxide (NO)	Arginine metabolism catalyzed by iNOS	Impairs mitochondrial function	Induces T cell apoptosis; Inhibits T cell proliferation and recruitment	(48, 49)
Polyamines	Arginine hydrolysis by Arginase 1 (ARG1)	Epigenetic modifications and translation regulation	Promotes Treg differentiation; Inhibits CD8^+^ T cell activation and functionality	(49, 50)

It is crucial to note that, while many of the immunometabolic mechanisms previously discussed are common across various solid tumors, gastric cancer should not be simplistically categorized as merely a hypoxic and glycolytic malignancy. Instead, its immunometabolic landscape is influenced by several disease-specific contextual factors that are either particularly pronounced or uniquely configured in this tumor type. First, gastric carcinogenesis typically occurs within a chronic inflammatory mucosal environment associated with Helicobacter pylori infection and broader gastric microbial dysbiosis. This context is particularly significant for gastric cancer, as persistent interactions between the host and microbiota can affect epithelial transformation, inflammatory signaling, nutrient competition, and local immune dynamics throughout the multistep process of tumorigenesis. Consequently, these factors serve to differentiate gastric cancer from many non-gastrointestinal solid tumors that do not experience such a sustained mucosal-microbial context (35–38).

Second, gastric cancer encompasses biologically distinct molecular subtypes that exhibit varying immune-metabolic contexts. Notably, Epstein-Barr virus-associated gastric cancer (EBVaGC) is increasingly acknowledged as an immunologically unique entity, characterized by an immune-rich or “immune-hot” phenotype, significant lymphoid infiltration, and pronounced immune-regulatory signaling, including features related to PD-L1 (38, 39). Consequently, the metabolic mechanisms underlying immune evasion in gastric cancer are unlikely to be consistent across different subtypes.

Third, recent studies on gastric cancer have underscored the significance of metabolic pathways that are intricately connected to local immune suppression. For instance, lactate accumulation in gastric cancer serves not only as a by-product of tumor glycolysis but also as an active immunoregulatory signal. A recent study demonstrated that lactate/GPR81 signaling facilitates the recruitment of regulatory T-cells via CX3CL1, thereby contributing to immune resistance in highly glycolytic gastric cancer (19). Concurrently, alterations in lipid metabolism have emerged as another critical feature relevant to gastric cancer, as dysregulated lipid metabolism can reshape the tumor immune microenvironment by influencing immune cell differentiation and function (35, 38, 40).

Finally, single-cell sequencing technology has provided deeper insights into the cellular heterogeneity and metabolic characteristics of the TME in gastric cancer. Specifically, this technology elucidates: a) the intricate interaction networks among cells within the TME of gastric cancer. For instance, it has been observed that LOX^+^ fibroblasts and M2-type macrophages are prevalent in gastric cancer tissues, interacting via the IL-6-IL6R signaling pathway to establish an immunosuppressive phenotype (41). b) the precise mechanisms underlying metabolic reprogramming in gastric cancer. By integrating single-cell RNA sequencing with spatial transcriptomics, researchers have identified that C1 NDUFAB1^+^ subtype cells exhibit high proliferative activity, metabolic reprogramming capabilities, and immune evasion characteristics (42). Furthermore, the study suggests that the state of cancer stem cell-like/epithelial-mesenchymal transition (EMT) cells in gastric cancer may be influenced by the interactions and metabolic reprogramming between cancer cells and macrophages (43). c) the heterogeneity among various molecular subtypes of gastric cancer and their association with metabolic reprogramming. For instance, research has demonstrated that patients with distinct molecular subtypes of gastric cancer exhibit significant variations in cellular composition and metabolic characteristics. This heterogeneity has potential implications for patient prognosis and treatment response (44, 45). For instance, gastric cancers classified as high metabolic subtypes are predominantly characterized by epithelial cell dominance, whereas those classified as low metabolic subtypes display substantial immune cell infiltration (46).

These studies suggest that although gastric cancer shares the core immune metabolism mechanism with other solid tumors, its biological explanation should be rooted in the unique gastric background caused by chronic mucosal inflammation, microbial community-related disturbance and subtype heterogeneity.

## 3 Nutrient competition serves as a driving force for immunosuppression

Within the gastric cancer TME, aberrant vascular hypoperfusion and heightened tumor cell metabolic activity create nutritional limitation. Here, nutrients function as the “fourth signal” in immune regulation, wherein preferential acquisition of glucose, amino acids, and lipids confers competitive advantage in functional differentiation (47). Consequently, nutrient competition is not merely a passive outcome but an active, modifiable driver of immunosuppression (48, 49).

### 3.1 Competition for glucose and the advantages of glycolysis

Glucose serves as a crucial carbon source, facilitating the rapid proliferation of T cells, as well as the synthesis of cytotoxic molecules and the secretion of cytokines. Upon activation, CD8^+^ T cells and Th1 cells typically upregulate glycolysis to swiftly satisfy the bioenergetic and biosynthetic demands required for their function (47, 50). Conversely, gastric cancer cells frequently exhibit a high glycolysis-high glucose uptake phenotype. By upregulating key regulators of glucose transport and glycolysis, these cancer cells preferentially sequester local glucose, thereby accelerating its consumption and creating a persistent low-glucose environment within the TME (35, 50–52). This competitive glucose consumption restricts the glucose metabolism of T cells. Furthermore, tumor cells exacerbate the suppression of T cell function by secreting metabolites such as lactic acid, which diminishes T cell efficacy under low-glucose conditions (53).

It is worth emphasizing that the selection pressure of nutrient competition will change the structure of the immune cell community. Tregs has more metabolic flexibility in energy acquisition, tends to use oxidative phosphorylation and fatty acid oxidation, and can limit excessive glycolysis dependence through FOXP3-related programs, thus maintaining survival and immunosuppression in low-sugar TME (54, 55). Similarly, the metabolic reprogramming of some tumor-associated macrophages (TAMs) enables them to obtain energy through oxidative phosphorylation (OXPHOS) and fatty acid oxidation (FAO) under the conditions of hypoxia and nutritional deficiency, thus supporting their tumor-promoting function (56, 57). Therefore, glucose competition not only “weakens effector T cells” but also “screens and enriches” immunosuppressed cells, making immunosuppression evolve from a transient event to a sustainable steady state (47, 49).

The immunological implications of glucose competition extend beyond mere substrate deprivation; they encompass the intricate transformation of metabolic dominance into suppressive signaling pathways. Recent research suggests that nutrients function as a “signal 4” in T-cell immunity, indicating that diminished glucose availability directly impacts T-cell activation, differentiation, and functional efficacy through mechanisms of nutrient sensing and metabolic programming (47). In the context of gastric cancer, elevated glycolytic flux not only limits glucose availability for effector lymphocytes but also facilitates lactate accumulation, which exacerbates immune dysfunction. Furthermore, MCT4-mediated lactate export has been implicated in metastasis-related immune remodeling in gastric cancer, further supporting the concept that glucose competition and lactate-driven immunosuppression are mechanistically integrated events rather than parallel phenomena (58). Therefore, glucose competition should be understood as a dual mechanism involving both the deprivation of glycolytic fuel for effector immune cells and the establishment of a lactate-dominant suppressive signaling network.

### 3.2 The metabolic limitation of glutamine and nitrogen

Glutamine serves as a critical intermediary between carbon and nitrogen metabolism, playing a dual role in the anabolism of the tricarboxylic acid cycle and acting as a vital nitrogen source and reducing agent for the synthesis of nucleotides, nonessential amino acids, and glutathione. Numerous tumors, including gastric cancer, exhibit a phenomenon referred to as glutamine dependence or glutamine addiction. This dependence is exacerbated by the reduced availability of glutamine within the TME, which is facilitated by enhanced transport mechanisms and the activation of glutamine catabolism to meet the demands of rapid cellular proliferation and antioxidant requirements (54, 59). In the context of T cells, glutamine functions not only as a nitrogen source essential for proliferation but also as a crucial substrate for sustaining biosynthetic processes. A glutamine deficiency can impair the clonal expansion and functional maintenance of activated T cells, leading to a shift in their metabolism toward a less efficient or stressed state (54, 59).

Furthermore, α-ketoglutaric acid (α-KG), a product of glutamine catabolism, serves as a cofactor for various dioxygenases and plays a role in histone demethylation, thereby influencing the inflammatory response and the polarization of macrophages (60, 61). Within the TME of gastric cancer, the competitive uptake of glutamine by tumor cells, coupled with hypoxic and acidic conditions, may predispose myeloid cells to adopt an immunosuppressive or tissue repair-like phenotype, thereby reinforcing the immunosuppressive feedback loop (62, 63).

Beyond serving as a biosynthetic substrate, glutamine functions as a critical regulator of immune-cell fate within metabolically stressed tumors. Mechanistically, glutamine deprivation restricts anaplerosis, nucleotide synthesis, and redox balance in activated T cells, thereby limiting sustained proliferation and cytokine production. Concurrently, glutamine-directed metabolic rewiring may conversely favor suppressive myeloid-cell programs. Experimental evidence has shown that targeting glutamine metabolism can reshape the tumor microenvironment by modulating suppressive myeloid cells, reducing IDO-related signaling, and enhancing tumor-specific immunity, indicating that glutamine metabolism occupies a nodal position between nutrient allocation and immune regulation (62). More recent studies have further emphasized that glutamine competition is not merely a matter of tumor cell addiction, but rather a dynamic process that redistributes metabolic privilege among tumor cells and immune subsets, thereby influencing whether the TME supports effector immunity or myeloid-dominant suppression (59). Consequently, the restriction of glutamine in gastric cancer could be more accurately understood as a dual mechanism that concurrently diminishes the biosynthetic competence of T-cells and promotes the persistence of suppressive myeloid-cell states.

### 3.3 Tryptophan depletion and immunomodulatory metabolites

Tryptophan, an essential amino acid, exhibits a characteristic enzyme-metabolite-receptor axis within the TME. In the case of gastric cancer, both cancer cells and TAMs/MDSCs can upregulate IDO1, catalyzing the conversion of tryptophan to kynurenine. This process leads to local depletion of tryptophan and accumulation of kynurenine (64, 65). This axis impairs anti-tumor immunity through two synergistic mechanisms: firstly, tryptophan deficiency induces amino acid stress, thereby restricting T cell proliferation and diminishing the expression of effector molecules; secondly, kynurenine acts as an endogenous ligand for the aryl hydrocarbon receptor (AHR), activating AHR signaling, which inhibits effector T cells, promotes regulatory T cell (Treg) differentiation, and concurrently enhances the immunosuppressive properties of myeloid cells (24, 25). In the context of gastric cancer, the tryptophan-kynurenine axis may also interact with tumor-associated inflammation, hypoxia, and metabolic acidification, thereby establishing a more stubborn immunosuppressive niche (25).

It is noteworthy that the immunological impact of this pathway cannot be fully explained by tryptophan depletion alone. Recent mechanistic investigations have revealed that tryptophan deficiency itself can sensitize the AHR pathway by upregulating AHR expression and facilitating GCN2/LAT1-mediated kynurenine uptake. This, in turn, significantly enhances the capacity of kynurenine to activate tolerance-associated transcriptional programs and promote Tregs differentiation (24). Consequently, substrate depletion and metabolite signaling do not function independently; rather, they interact synergistically to potentiate immunosuppression. In the context of gastric cancer, this effect may be further intensified by the involvement of tumor-infiltrating monocytic MDSCs, which have been shown to play a significant role in establishing an immunosuppressive and ICIs-resistant TME (66).

### 3.4 Arginine depletion and myeloid cell-driven immunosuppression

The activation, proliferation, and functional maintenance of effector T cells necessitate adequate intracellular nucleotide and protein synthesis, with arginine serving as a critical substrate. Tumor-infiltrating mononuclear myeloid-derived suppressor cells (TI-M-MDSCs) exhibit elevated expression of immunosuppressive genes within gastric cancer tissues, and their degree of enrichment is significantly associated with poor patient prognosis (66). Furthermore, MDSCs are intricately linked to the progression of gastric cancer and the mechanisms of immune evasion, forming a complex immunosuppressive network through interactions with TAMs and other immune cells (67). For instance, within the TME of gastric cancer, MDSCs and M2-like TAMs frequently deplete arginine by overexpressing arginase 1 (ARG1) and inducible nitric oxide synthase (iNOS) (32). ARG1 catalyzes the conversion of arginine into ornithine and polyamines, whereas iNOS converts arginine into nitric oxide (NO) and citrulline, resulting in a reduction of local effective arginine concentration and an increase in immunosuppressive metabolites (32). These metabolites exert potent immunomodulatory effects.

In mechanistic terms, arginine metabolism exerts immunosuppressive effects through both substrate depletion and the generation of downstream metabolites. Arginine depletion restricts the biosynthetic and signaling processes essential for T-cell proliferation and effector differentiation. Concurrently, the products of ARG1/iNOS activity, such as nitric oxide and polyamines, further inhibit T-cell receptor signaling, compromise mitochondrial and metabolic fitness, and facilitate the stabilization of suppressive immune phenotypes. Within this framework, myeloid cells do not merely compete for arginine but actively transform arginine metabolism into a multifunctional suppressive axis. This concept is corroborated by mounting evidence indicating that arginase-dependent pathways in tumors are pivotal components of myeloid-mediated immune suppression and are increasingly being investigated as therapeutic targets for the restoration of antitumor immunity (32). Consequently, in the TME of gastric cancer, arginine competition should be understood as a myeloid-centered metabolic checkpoint that connects nutrient depletion with the accumulation of suppressive metabolites and immune dysfunction.

### 3.5 Imbalance of lipid availability: scarcity, overload, and lipotoxicity

Unlike the scarcity of glucose and amino acids, lipids within the TME frequently exhibit an availability imbalance. Certain micro-regions experience a deficiency of accessible fatty acids for effector T cells, while others are saturated with lipids due to factors such as necrosis, obesity-related metabolic abnormalities, or heightened uptake by tumor and myeloid cells (68). In macrophages, particularly TAMs, excessive lipid intake promotes a shift towards the M2 phenotype, a process associated with the activation of the peroxisome proliferator-activated receptor (PPAR) signaling pathway (57, 69). For effector T cells and NK cells, exposure to elevated levels of free fatty acids, particularly saturated fatty acids, results in abnormal lipid accumulation within the cells. This accumulation induces endoplasmic reticulum stress and mitochondrial dysfunction, collectively termed lipotoxicity, ultimately impairing the cells’ effector functions and survival (70). Notably, oxidized lipids are internalized via the scavenger receptor CD36, leading to lipid peroxidation that directly compromises the effector function of CD8^+^ T cells. This process represents a critical mechanism underlying immune dysfunction due to lipid competition (71). Consequently, conceptualizing lipid metabolism as an availability imbalance rather than merely an increase or decrease in lipid levels offers a more nuanced understanding of its bidirectional immunological effects. This perspective can inform more precise target selection, such as inhibiting lipid uptake, enhancing lipid catabolism, or improving lipid quality.

From a mechanistic perspective, lipid competition differs from glucose and amino acid competition in that its immunological consequences depend not only on quantity but also on lipid composition and cellular handling. In particular, oxidized lipids can be internalized through CD36, resulting in intracellular lipid peroxidation and progressive dysfunction of intratumoral CD8^+^ T cells (71). This finding indicates that excessive lipid availability does not necessarily support immune-cell fitness, but may instead promote a form of metabolic toxicity that selectively weakens antitumor effector cells. In parallel, lipid-derived signaling mediators such as PGE2 can reinforce suppressive programs in myeloid cells through EP2/EP4 receptors, thereby further stabilizing the immunosuppressive niche (30). Thus, lipid competition in gastric cancer should be understood not merely as a problem of scarcity or overload, but as a bidirectional process involving lipid uptake, oxidative stress, and lipid-mediated immune signaling, all of which shape the functional balance between effector and suppressive immune populations.

**Figure 1:**
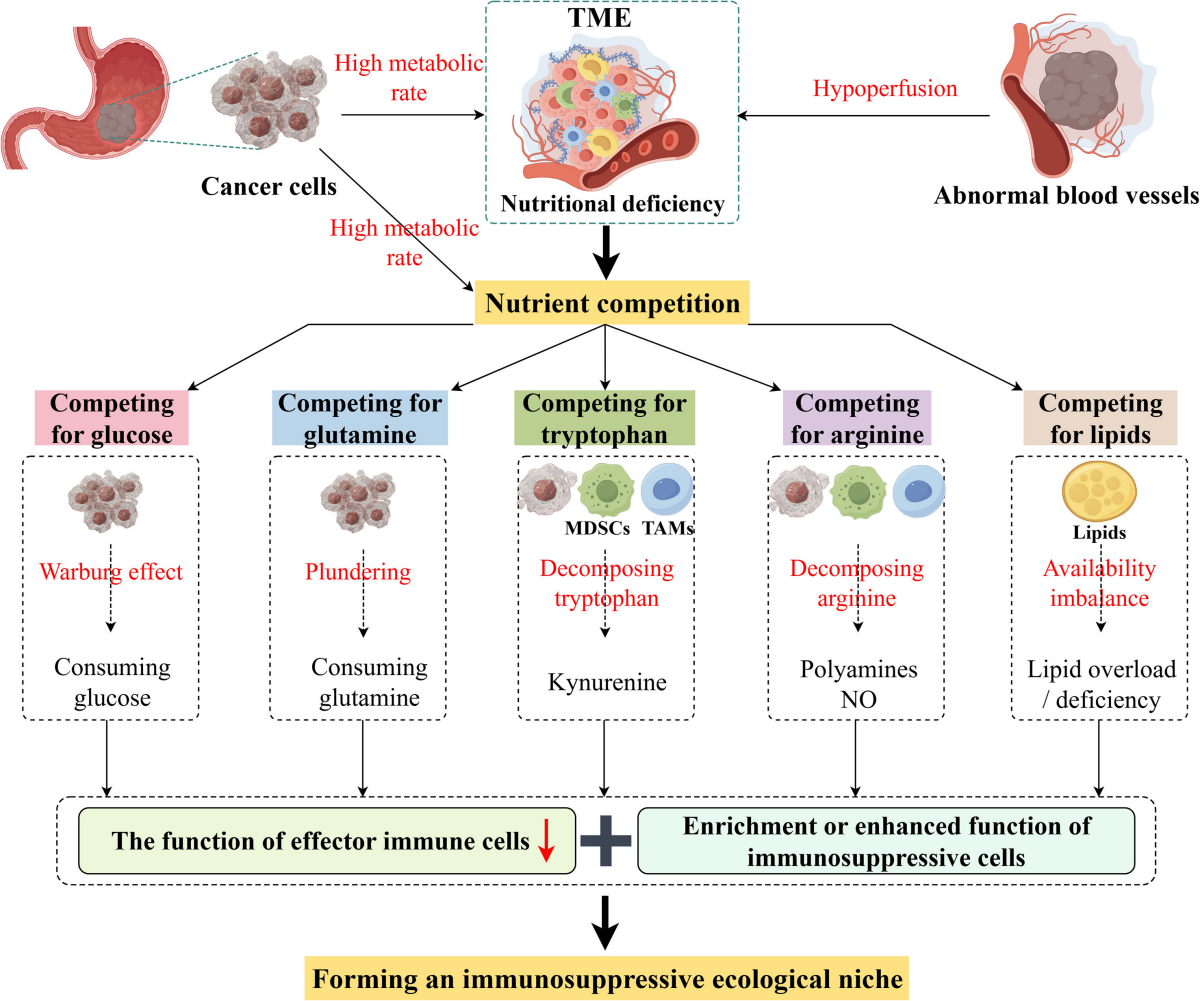
Schematic diagram illustrating nutrient competition-driven immunosuppression in the TME of gastric cancer. This image depicts the core mechanism by which nutrient competition in the gastric cancer tumor microenvironment (TME) drives immunosuppression. Upstream, two factors induce nutritional deficiency in the TME: the high metabolic rate of gastric cancer cells, and hypoperfusion caused by abnormal blood vessels; this deficiency further triggers intercellular nutrient competition. Five key nutrient competition axes are included in this diagram: 1) glucose competition: gastric cancer cells utilize the Warburg effect (aerobic glycolysis) to consume large amounts of glucose; 2) glutamine competition: cancer cells plunder glutamine in the TME; 3) tryptophan competition: Myeloid-derived suppressor cells (MDSCs) and tumor-associated macrophages (TAMs) decompose tryptophan to produce kynurenine; 4) arginine competition: MDSCs/TAMs decompose arginine, generating polyamines and nitric oxide (NO); 5) lipid competition: Lipid availability imbalance (overload or deficiency) occurs in the TME. These competition processes synergistically lead to two critical consequences: functional impairment of effector immune cells, and enrichment/enhanced function of immunosuppressive cells. Ultimately, these changes converge to form an immunosuppressive ecological niche in the gastric cancer TME, which promotes tumor immune escape. This image was drawn by Figdraw (a drawing platform).

In the TME of gastric cancer, nutrient competition transcends a mere contest for intercellular resources, emerging as a central mechanism that orchestrates immunosuppression through the precise regulation of metabolic homeostasis and the functional fate of immune cells. This competition is not an isolated phenomenon; rather, it centers around five critical nutrients—glucose, glutamine, tryptophan, arginine, and lipids—culminating in the establishment of a microenvironmental niche that is unfavorable to anti-tumor immunity (**Figure 1**).

## 4 The metabolic crosstalk network for maintaining the immunosuppressive niche

Nutrient competition reshapes the metabolic composition of the TME. These metabolites are not merely “waste products” but serve as intercellular messengers that can diffuse, be transported, and be incorporated into extracellular vesicles (EVs). These EVs facilitate communication between tumor nests, stromal regions, and lymphoid structures, thereby establishing a chronic and self-perpetuating immunosuppressive niche over time (72). These metabolites share common characteristics: firstly, they directly disrupt the energy and redox homeostasis of effector immune cells (73); secondly, they reprogram the fate of immune cells through receptor-mediated signaling pathways or epigenetic mechanisms, thereby transforming nutritional stress into immune tolerance (74).

### 4.1 The lactic acid-acidosis axis

In the TME of gastric cancer, the accumulation of lactic acid is recognized as a critical factor in fostering the development of an immunosuppressive milieu. Lactic acid engages with specific receptors to activate downstream signaling pathways, thereby remodeling the TME and facilitating tumor progression (75). Furthermore, the buildup of lactic acid results in the acidification of the TME, which impairs the normal function of immune cells and enhances tumor immune evasion (76, 77).

**Figure 2:**
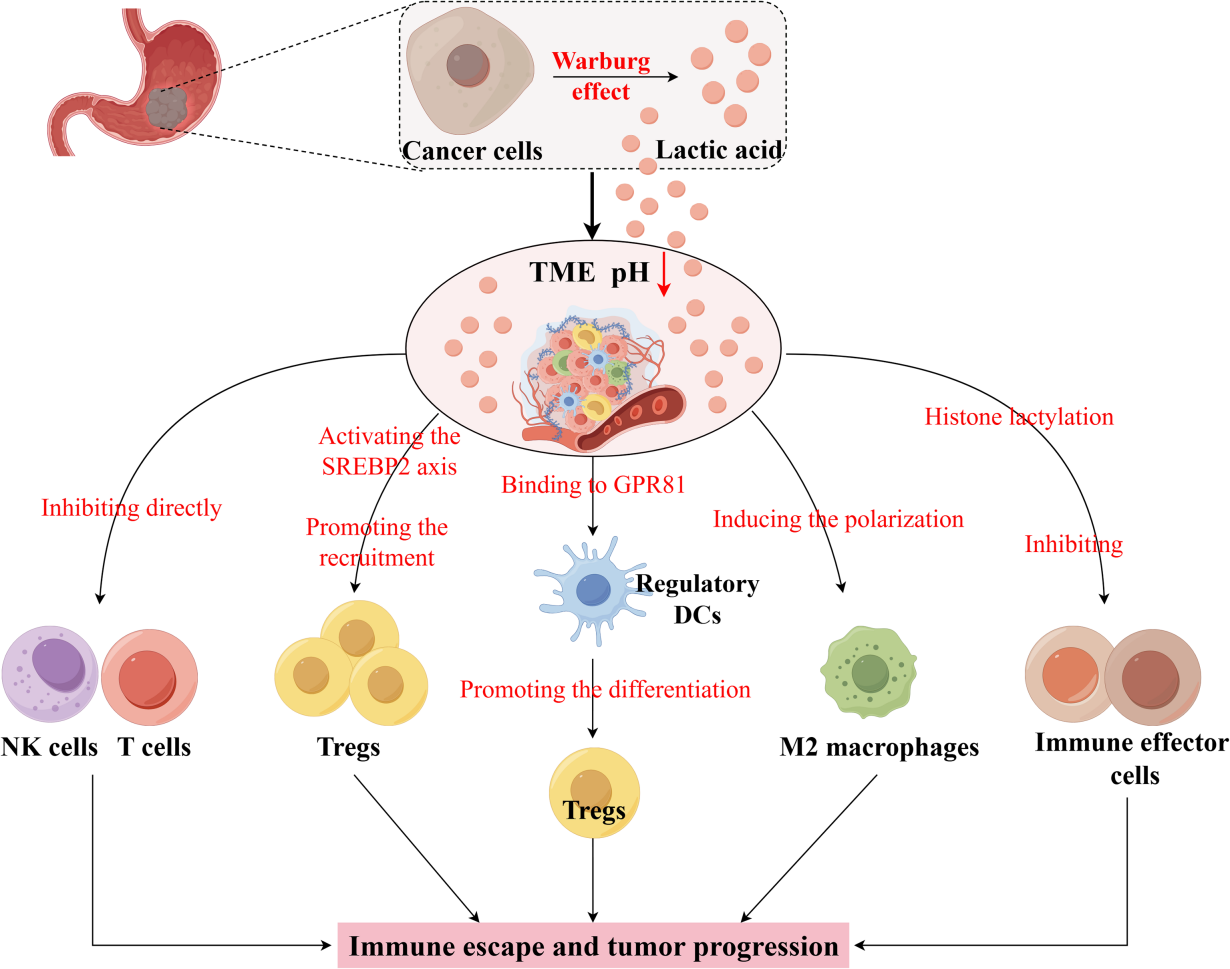
The mechanism of lactate-acidosis axis-mediated immunosuppression in the TME of gastric cancer. In the upper left quadrant of the figure, gastric cancer cells produce substantial amounts of lactic acid via the Warburg effect, subsequently releasing it into the tumor microenvironment (TME), thereby directly contributing to its acidification. This acidic milieu modulates the functions of various immune cells, including the direct inhibition of NK cells and T cells. Furthermore, it activates the sterol regulatory element-binding protein 2 (SREBP2) axis to recruit regulatory T cells (Tregs), interacts with the G-protein-coupled receptor 81 (GPR81) to facilitate Treg differentiation, and induces M2 macrophage polarization through histone lactylation, while inhibiting immune effector T cells. Collectively, these alterations in immune cell functions orchestrate the formation of an immunosuppressive microenvironment, ultimately facilitating immune evasion and promoting the progression of gastric cancer. This image was drawn by Figdraw (a drawing platform).

In the TME of gastric cancer, the lactic acid-acidosis axis significantly contributes to the establishment of an immunosuppressive milieu through multiple mechanisms. Firstly, the presence of lactic acid and the resultant acidic environment attenuates the effector functions of NK cells and T cells, manifesting as reduced cytotoxicity and proliferative capacity (16, 17). Secondly, lactic acid produced by tumor cells can induce a tolerant or regulatory phenotype in dendritic cells by modulating the lipid metabolism transcriptional program, specifically the SREBP2 axis. This modulation inhibits antigen cross-presentation and promotes the differentiation of Tregs, thereby shifting the pivotal immune response initiation node towards immunosuppression (18). Thirdly, lactic acid further suppresses the function of CD8^+^ T cells by inducing the polarization of M2-type macrophages, contributing to the formation of an immunosuppressive TME (20). Fourthly, as a critical mechanism of epigenetic regulation, lactate influences gene transcription by modulating the modification of histone and non-histone proteins, thereby promoting the establishment of an immunosuppressive microenvironment (78). **Figure 2** illustrates this cascade regulatory network, encompassing lactic acid production, efflux, signal transduction, and the remodeling of immune cell function.

### 4.2 The ATP-adenosine axis

Tumor cell stress or death leads to the release of large amounts of adenosine triphosphate (ATP) into the extracellular space. Within the TME, extracellular ATP (eATP) is swiftly hydrolyzed by a series of ectonucleotidases: initially, CD39 converts ATP to AMP, followed by the conversion of AMP to adenosine by CD73 (79). The resultant elevated adenosine concentration engages the adenosine A2A receptor (ADORA2A) on immune cell surfaces with high affinity, thereby activating the intracellular cAMP-PKA signaling pathway. This activation significantly suppresses the proliferation, cytokine production, and cytotoxic functions of effector immune cells, such as T cells and NK cells, while concurrently enhancing the immunosuppressive activity of Tregs (21–23). This phenomenon is analogous to a soluble, metabolically derived immune checkpoint. Adenosine, an endogenous purine nucleoside, modulates immune cell function by interacting with A2A and A2B receptors, particularly within the TME. The accumulation of adenosine is regarded as a significant mechanism contributing to tumor immune evasion (80, 81).

Recently, the exploration of antitumor therapies targeting the ATP-adenosine axis has emerged as a significant area of research within the field of tumor immunotherapy. The ATP-adenosine axis has been identified as a crucial therapeutic target due to its role in modulating immunosuppression and facilitating tumor progression within the TME. For instance, research has demonstrated that the blockade of the adenosine A2A receptor can restore the cytotoxic function of T cells in tumors characterized by metabolic dysregulation, thereby enhancing the efficacy of immunotherapeutic interventions (82). Furthermore, the inhibition of the adenosine pathway has been shown to augment the effectiveness of PD-1/PD-L1 immune checkpoint inhibitors, with combination therapies exhibiting superior antitumor outcomes (83).

### 4.3 The tryptophan-kynurenine-AHR axis

The tryptophan-kynurenine-AHR axis is critically involved in the development of immunosuppression within the TME of gastric cancer. Kynurenine enzymes in the tryptophan metabolic pathway, such as indoleamine 2,3-dioxygenase 1 (IDO1) and tryptophan 2,3-dioxygenase (TDO), facilitate the activation of the aryl hydrocarbon receptor (AHR) by converting tryptophan into kynurenine, thereby exerting an immunosuppressive effect in the TME (26). Research indicates that AHR activation can promote immune tolerance by modulating the differentiation and function of immune cells. For instance, AHR activation has been shown to enhance the proliferation of Tregs and augment their immunosuppressive capabilities by upregulating the expression of PD-1 (27). This metabolic pathway not only depletes tryptophan but also directly suppresses the immune response through the interaction of kynurenine with AHR, thereby facilitating tumor immune evasion (84).

### 4.4 The arginine -NO/polyamine axis

In the TME of gastric cancer, arginine metabolism serves as the principal metabolic axis underpinning myeloid cell-mediated immunosuppression. TAMs and MDSCs exhibit elevated expression of ARG1 and iNOS, which deplete arginine in the microenvironment via two parallel pathways, thereby significantly inhibiting the function of effector T cells (33). iNOS catalyzes the conversion of arginine into NO, and elevated NO concentrations can impair T cell receptor signaling, disrupt metabolic pathways, and induce T cell apoptosis. Concurrently, ARG1 hydrolyzes arginine into ornithine, which subsequently facilitates polyamine synthesis (33). Polyamines not only support tumor cell proliferation but also directly inhibit T cell activation and promote the differentiation of Tregs (34). Furthermore, arginine depletion itself triggers a nutritional stress response in T cells, limiting their proliferation and cytokine production. This metabolic axis synergizes with conditions such as hypoxia and acidification, further reinforcing the immunosuppressive niche centered around myeloid cells, and providing a theoretical framework for the development of therapeutic strategies targeting ARG1/iNOS in combination with ICIs (85).

### 4.5 The lipid media axis

Lipids serve not only as energy substrates but also as crucial immunomodulatory agents through their derivatives. Cyclooxygenase-2 (COX-2) is frequently overexpressed in gastric cancer and catalyzes the conversion of arachidonic acid into prostaglandin H2 (PGH2), subsequently leading to the production of prostaglandin E2 (PGE2) (28). PGE2 exerts an inhibitory effect on various immune cells via its receptors (EP2/EP4), including: 1) inhibiting the maturation of dendritic cells (DCs) (29); 2) promoting the expansion and differentiation of MDSCs and enhancing their immunosuppressive properties (30); 3) suppressing the proliferation and cytotoxicity of T cells and NK cells (86, 87); and 4) upregulating the expression of PD-L1 on tumor cells and myeloid cells (31).

### 4.6 The network hub and channel crosstalk

Multiple metabolic inhibition pathways do not function in isolation; rather, they constitute a highly interconnected network structure by sharing common signaling hubs. Notably, mTORC1, HIF-1α, and AMPK are regarded as the central nodes (47–49) that integrate nutritional sensing, metabolic adaptation, and immune regulation. Nutrient deprivation and hypoxia collaboratively constrain the metabolism of effector T cells and the synthesis of effector molecules. In contrast, tumor cells and immunosuppressive myeloid cells exhibit enhanced glycolytic capabilities and facilitate the activation of immunosuppressive molecules and pathways driven by hypoxia-related mechanisms (48–50). Metabolites such as lactic acid, adenosine, and kynurenine further suppress T cell activation and anti-tumor responses through receptor-mediated signaling pathways (e.g., GPR81, A2A, AHR), establishing a network inhibition effect analogous to a metabolic checkpoint (18, 19, 79, 81). In the adenosine pathway, the metabolism of extracellular nucleotides (via CD39/CD73-adenosine) and A2A receptor signaling are recognized as critical metabolic checkpoints in tumor immunosuppression (79, 81). The accumulation of kynurenine, resulting from tryptophan catabolism, can enhance the sensitivity of the AHR pathway, facilitate regulatory immune processes, and stabilize the immunosuppressive niche (24, 88). Furthermore, metabolic signals can modulate the state of immune cells at the epigenetic level, thereby solidifying the inhibitory phenotype and enhancing the stability of the network (48, 49). This multifaceted crosstalk renders single-target interventions susceptible to compensatory mechanisms. Therefore, the joint regulation of metabolic and immune signals at the network hub level may be a pivotal strategy for reshaping the immune microenvironment in gastric cancer and improving the efficacy of immunotherapy.

To thoroughly understand the integrated metabolic network within the TME of gastric cancer, it is essential to elucidate the regulatory interactions among the three principal signaling nodes: AMPK, mTORC1, and HIF-1α. Under physiological conditions, AMPK, an energy sensor, is activated in response to nutrient deprivation or metabolic stress, indicated by an elevated AMP/ATP ratio, to conserve energy by directly inhibiting mTORC1 (89, 90). However, within the metabolically demanding TME of gastric cancer, tumor cells often dysregulate this axis to maintain aggressive proliferation. The aberrant activation of the PI3K/AKT/mTORC1 pathway, frequently accompanied by impairment of LKB1/AMPK signaling, leads to the persistent translation and stabilization of HIF-1α, even in normoxic conditions (91, 92). Consequently, HIF-1α functions as the key transcriptional regulator that integrates various metabolic pathways, thereby transforming the TME into an immunosuppressive environment (93). Crucially, HIF-1α orchestrates the cross-activation of both the lactate and adenosine immunosuppressive pathways. Specifically, HIF-1α enhances the expression of key glycolytic enzymes, such as hexokinase 2 (HK2), pyruvate kinase M2 (PKM2), and lactate dehydrogenase A (LDHA), along with the monocarboxylate transporter 4 (MCT4), thereby promoting extensive aerobic glycolysis and the subsequent release of lactic acid into the TME (94–97). Simultaneously, HIF-1α facilitates the purinergic signaling cascade by upregulating ectonucleotidases CD39 and CD73 (98, 99). These enzymes rapidly degrade the extracellular ATP released during cellular stress into adenosine, which has immunosuppressive effects (100). This results in a significant metabolic synergy: the concurrent accumulation of lactate and adenosine severely impairs the activation of NK cells and effector T cells, while driving the polarization of TAMs towards an M2 phenotype (11, 101, 102). Furthermore, lactate functions as a signaling molecule in a positive feedback loop, enhancing histone lactylation, which in turn stabilizes HIF-1α and mTORC1 signaling, thus perpetually increasing lactate and adenosine production (103, 104). Ultimately, the AMPK-mTORC1-HIF-1α tripartite hub functions as the central engine of the metabolic network, dynamically coordinating glycolysis and purine metabolism to solidify immune exclusion in gastric cancer. The central axis comprising mTORC1, HIF-1α, and AMPK synergistically modulates various immunosuppressive metabolic pathways, facilitating the amplification of immunosuppression at the network level within the TME of gastric cancer. This hub-mediated pathway synergy mechanism indicates that immunosuppression in the TME is not attributable to a singular metabolic pathway. Instead, it is achieved through the cross-regulation of multiple pathways via the core hub. Modulating the signaling pathways of AMPK, mTORC1, and HIF-1α may offer novel insights and strategies for the treatment of gastric cancer.

## 5 Therapeutic strategies of targeted metabolic reprogramming for gastric cancer

The elucidation of the intricate network involving nutrient competition-metabolic crosstalk-immunosuppression within the immunosuppressive microenvironment of gastric cancer has broadened the scope of metabolic interventions. These interventions have evolved from solely inhibiting tumor proliferation to also alleviating immune metabolic constraints and restoring effective immunity (5, 105–107). This advancement provides a theoretical foundation for the development of novel metabolic intervention strategies. The application of these strategies is anticipated to enhance the efficacy of immunotherapy by alleviating immune metabolic restrictions and reinstating effective immune function. In light of this mechanism, a diverse array of drugs targeting metabolic reprogramming has been developed in recent years. We have compiled a selection of representative drugs and their mechanisms of action, focusing on the metabolic reprogramming of gastric cancer (**Table 2**).

**Table 2:** Some representative drugs and action mechanism of metabolic reprogramming targeting gastric cancer.

Therapeutic target	Example agents	Mechanism of action	References
Glycolysis Pathway	2-DG; Barasertib; LicA	Inhibits HK2 or LDHA activity; blocks glucose utilization and reduces lactate production to alleviate T cell glucose deprivation.	(94–96)
Lactate Transport	Syrosingopine	Inhibits MCT1/MCT4 transporters; prevents lactate excretion, and improves the pH value of the microenvironment.	(97, 98)
Tryptophan-Kynurenine Axis	Epacadostat; Indoximod	Inhibits IDO1 enzymatic activity; reduces immunosuppressive Kynurenine levels and relieves AHR-mediated T cell suppression.	(67, 91, 99)
Arginine Metabolism	CB-1158; OATD-02	Inhibits Arginase; prevents arginine depletion in the TME, thereby restoring CD3ζ expression and T cell proliferation.	(88, 100)
Glutamine Metabolism	CB-839; V-9302	Blocks glutamine uptake or catabolism; induces oxidative stress and apoptosis in tumor cells.	(101, 102)
Lipid Metabolism	Celecoxib	Inhibits COX-2; decreases PGE2 synthesis, hindering the induction and recruitment of MDSCs and Tregs.	(103, 104)
Adenosine Signaling	AB680 (CD73 inhibitor); AB598 (CD39 inhibitor)	Blocks the conversion of extracellular ATP to Adenosine; alleviates A2A receptor-mediated signaling that inhibits T cell cytotoxicity.	(105–107)

Most agents listed are currently in preclinical development or early-phase clinical trials (Phase I/II) for solid tumors. Celecoxib is FDA-approved for non-oncology indications but is under investigation for gastric cancer utility.

2-DG, 2-Deoxyglucose; LicA, Licochalcone A; HK2, Hexokinase 2; LDHA, Lactate Dehydrogenase A; MCT, Monocarboxylate Transporter; AHR, Aryl Hydrocarbon Receptor; PGE2, Prostaglandin E2; and MDSCs, Myeloid-derived suppressor cells.

### 5.1 Targeting glycolysis and the lactic acid metabolism axis

#### 5.1.1 Glycolysis inhibitors

In recent years, there has been significant interest in the use of glycolysis inhibitors for the treatment of gastric cancer. Glycolysis represents a crucial pathway in the reprogramming of energy metabolism within cancer cells, particularly in gastric cancer, where its upregulation is closely associated with tumor malignancy and poor prognosis (122, 123). Consequently, inhibitors targeting the glycolytic pathway are regarded as potential therapeutic strategies.

In gastric cancer, protein phosphatase 2A (PP2A) plays a crucial role in inhibiting glycolysis by attenuating the c-Myc signaling pathway (124). A reduction in PP2A activity has been associated with increased proliferation and glycolytic capacity in gastric cancer cells. Conversely, pharmacological activation of PP2A has been shown to significantly diminish both proliferation and glycolysis in these cells (124). Furthermore, barasertib, an Aurora kinase B inhibitor, has been observed to decrease glycolytic activity in gastric cancer cells by downregulating the expression of the glucose transporter GLUT1 and LDHA (108).

Natural compounds exhibit potential in the inhibition of glycolysis in gastric cancer. Research has demonstrated that glycyrrhiza chalcone A (LicA) suppresses tumor glycolysis mediated by HK2 through the downregulation of the Akt signaling pathway, thereby inhibiting the proliferation of gastric cancer cells and inducing apoptosis (109). Furthermore, the flavonoid catechin has been shown to enhance the sensitivity of gastric cancer cells to 5-fluorouracil by inhibiting the activity of LDHA (110).

#### 5.1.2 Lactate transporter inhibitors

Lactic acid, the terminal product of glycolysis, functions not only as a metabolic byproduct but also plays a critical role in the proliferation and invasion of tumor cells. Research indicates that lactic acid facilitates metabolic reprogramming and the malignant progression of tumors through the activity of monocarboxylic acid transporters (MCTs) (125). Consequently, targeting lactate transporters presents a promising anti-cancer strategy. In gastric cancer, MCT1 and MCT4 serve as the primary lactate transporters, and their inhibition, particularly through agents such as syrosingopine in combination with metformin, can disrupt glycolysis and induce cell death by depleting intracellular NAD+ (111). Furthermore, the inhibition of MCT1 can impede the lactic acid-induced activation of HIF-1, thereby suppressing tumor angiogenesis (112).

### 5.2 Targeting the amino acid metabolism network

#### 5.2.1 Blocking the tryptophan-kynurenine pathway

The potential therapeutic application of the tryptophan-kynurenine pathway in gastric cancer treatment has garnered increasing scholarly interest. In gastric cancer, IDO1 catalyzes the conversion of tryptophan into kynurenine, a process that may contribute to immunosuppression within the tumor microenvironment and facilitate tumor proliferation and metastasis (64). This mechanism is particularly pronounced in invasive gastric cancer subtypes, such as signet ring cell carcinoma (SRCC), which are characterized by elevated IDO1 expression and reduced tryptophan levels (64). Furthermore, metabolites of the IDO1 and kynurenine pathway can activate the PI3K-Akt signaling pathway, thereby promoting cancer cell proliferation and inhibiting apoptosis (113).

Consequently, the inhibition of IDO1 and the kynurenine pathway may represent a promising therapeutic strategy, capable of suppressing tumor proliferation and augmenting the anti-tumor immune response by obstructing these signaling pathways. Furthermore, within the context of therapeutic strategies, some researchers have proposed that concurrently inhibiting the rate-limiting enzymes IDO1 and TDO2 within this pathway could optimize the therapeutic efficacy of kynurenine pathway inhibition (88).

#### 5.2.2 Regulating the arginine metabolism

Arginase plays a critical role in inhibiting T cell proliferation by depleting L-arginine within the TME, thereby facilitating tumor growth and immune evasion. Consequently, arginase inhibitors have emerged as a promising strategy for anticancer therapy. OATD-02 represents a novel dual arginase (ARG1/ARG2) inhibitor that effectively suppresses arginase activity both intracellularly and extracellularly. This action restores L-arginine levels within the TME, reduces polyamine synthesis, and enhances the anticancer efficacy of immune cells (114). Empirical evidence indicates that OATD-02 significantly enhances the antitumor immune response in murine models, increases the infiltration of CD8^+^ T cells into tumors, and markedly improves therapeutic outcomes when used in conjunction with anti-PD-1 therapy (114). Furthermore, CB-1158, another potent arginase inhibitor, has been shown to significantly augment the infiltration of CD8^+^ T cells and NK cells into tumors by counteracting myeloid cell-mediated immune suppression, thereby bolstering the antitumor immune response (85).

#### 5.2.3 The intervention of the glutamine metabolism

Glutamine metabolism undergoes significant reprogramming in gastric cancer cells, emerging as a crucial energy source and biosynthetic precursor that supports cancer cell growth and survival (115). Consequently, targeting glutamine metabolism represents a promising therapeutic strategy to inhibit gastric cancer progression.

Firstly, the expression of glutamine transporters, such as ASCT2, is frequently up-regulated in gastric cancer cells, thereby promoting glutamine uptake and utilization. Studies have demonstrated that inhibiting the glutamine transporter ASCT2 or the enzyme glutamine synthetase (GS) effectively suppresses gastric cancer cell growth (115). Moreover, restricting glutamine availability can enhance the efficacy of conventional anticancer agents. For example, glutamine deprivation synergistically increases the inhibitory effect of cetuximab on gastric cancer cells (116). Secondly, inhibiting glutamine metabolism can induce cancer cell apoptosis and growth arrest through multiple mechanisms. For instance, it promotes oxidative stress and triggers autophagy, leading to enhanced cancer cell death (126, 127). Furthermore, interference with glutamine metabolism modulates key signaling pathways—including STAT3 and mTOR—that regulate cancer cell proliferation and survival (128, 129).

### 5.3 Targeting lipid metabolism remodeling

In gastric cancer, the reprogramming of lipid metabolism not only promotes cancer cell growth and survival but also impairs therapeutic efficacy (130). For example, PLA2G4A and ACHE modulate the endogenous lipid profile via the glycerophospholipid metabolism pathway, influencing treatment outcomes in platinum−resistant gastric cancer patients (131). Additionally, HKDC1 reprograms lipid metabolism by forming a ribonucleoprotein complex, thereby enhancing metastasis and conferring cisplatin resistance in gastric cancer (132).

Intervention strategies targeting lipid metabolism present promising avenues for the treatment of gastric cancer. For instance, the inhibition of lipid metabolism has been shown to enhance the efficacy of chemotherapeutic agents by inducing ferroptosis (133). Furthermore, natural compounds, including flavonoids and saponins, demonstrate potential in inhibiting the initiation and progression of gastrointestinal cancers through the modulation of lipid metabolism-related pathways (134). In summary, the regulation of lipid metabolism may serve not only as a therapeutic target for gastric cancer but also as a means to augment the overall effectiveness of treatment, particularly by enhancing the outcomes of immunotherapy (135).

### 5.4 Targeting the signal axis of immunosuppressive metabolites

#### 5.4.1 Blocking the ATP- adenosine axis

The ATP-adenosine axis, mediated by CD39 and CD73, plays a pivotal role in immune evasion and tumor progression in gastric cancer. Within the context of gastric cancer treatment, the adenosine signaling pathway has emerged as a novel immune checkpoint. It has been demonstrated to significantly impair the anti-tumor immune response by inhibiting the infiltration and function of CD8^+^ T cells and natural killer cells (136, 137). Research indicates that the inhibition of CD39 and/or CD73 can effectively counteract this immunosuppressive state, thereby enhancing the anti-tumor activity of immune cells (138, 139). Moreover, blocking these ectoenzymes reduces adenosine accumulation in the TME, thereby promoting immune cell infiltration and activation, and ultimately enhancing the efficacy of immunotherapy (140, 141).

Recent years have witnessed significant advances in the development of inhibitors targeting CD39 and CD73. For instance, AB680, a potent small-molecule CD73 inhibitor, has demonstrated promising anti-tumor efficacy in preclinical models and is currently undergoing clinical trials in combination with ICIs (119, 120). Similarly, the CD39 inhibitor AB598 has shown potential in preclinical studies to enhance anti-tumor immune responses by elevating extracellular ATP levels while reducing adenosine accumulation within tumors (121). Collectively, these findings underscore that targeting the CD39/CD73 axis can effectively potentiate immunotherapy and may offer novel therapeutic strategies for refractory malignancies such as gastric cancer (141).

#### 5.4.2 Inhibiting the COX-2/PGE2 axis

Cyclooxygenase-2 (COX-2) serves as a pivotal enzyme in the biochemical conversion of arachidonic acid to prostaglandin E2 (PGE2), which is frequently overexpressed in various malignancies, including gastric cancer, and is linked to unfavorable prognoses (28). PGE2 is instrumental in mediating immunosuppression and facilitating tumor progression via its receptors, EP2 and EP4, particularly in the pathogenesis and advancement of gastric cancer (142, 143). Studies indicate that PGE2 enhances tumor cell proliferation, migration, and invasion through EP2 and EP4 receptor-mediated signal transduction, while concurrently suppressing immune cell function (30, 144). Furthermore, PGE2 has been shown to promote the differentiation of MDSCs within TME through EP2 and EP4 receptors, thereby exacerbating immunosuppressive conditions (145, 146).

The administration of COX-2 inhibitors, such as Celecoxib, has been shown to substantially decrease the production of PGE2, thereby mitigating the immunosuppressive effects associated with tumors and augmenting the anti-tumor immune response (117, 118). Furthermore, dual inhibition of EP2 and EP4 receptors has demonstrated a significant enhancement in immune cell activity and a reduction in tumor burden, outperforming single receptor inhibition across various tumor models (147, 148).

**Figure 3:**
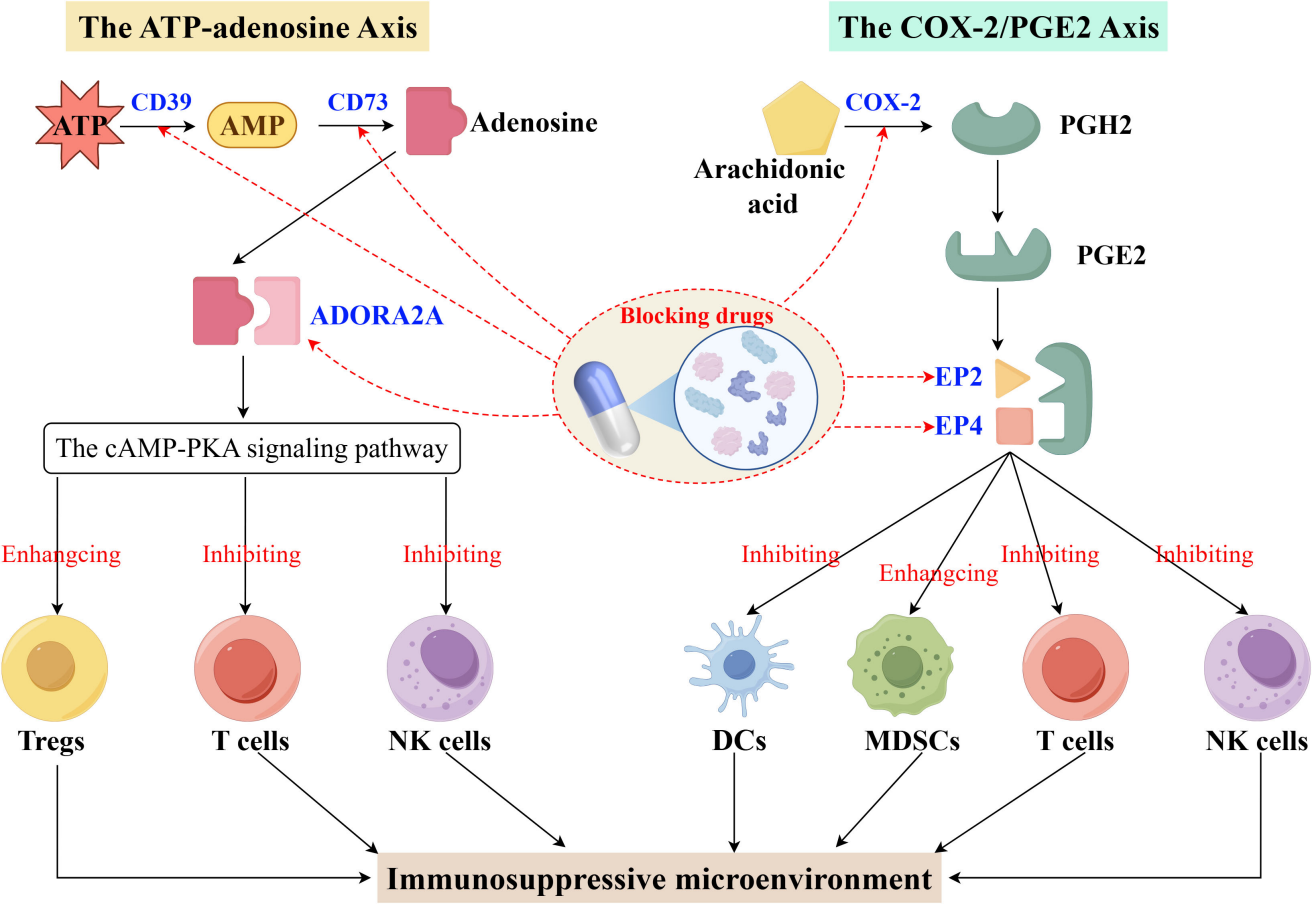
Immunosuppressive metabolite signaling axes and intervention targets in gastric cancer TME. Left: ATP-adenosine axis: ATP is catalyzed into AMP by CD39, and AMP is further hydrolyzed to form adenosine via CD73. After adenosine binds to the adenosine A2A receptor (ADORA2A), it activates the cAMP-PKA signaling pathway, which ultimately enhances the immunosuppressive function of regulatory T cells (Treg) while inhibiting the activity of cytotoxic T cells and natural killer (NK) cells. Right: COX-2/PGE2 axis: Arachidonic acid is catalyzed by cyclooxygenase-2 (COX-2) to generate the prostaglandin intermediate PGH2, which is eventually converted into prostaglandin E2 (PGE2). After PGE2 binds to the EP2/EP4 receptors on the surface of immune cells, it exerts immunomodulatory effects: inhibiting the maturation of dendritic cells (DCs), impairing the activity of cytotoxic T cells and NK cells, and simultaneously enhancing the immunosuppressive function of myeloid-derived suppressor cells (MDSCs). In the figure, “Blocking drugs” denotes the intervention agents targeting key nodes of the aforementioned signaling axes. The synergistic effects of these two signaling axes ultimately contribute to shaping the immunosuppressive microenvironment. This image was drawn by Figdraw (a drawing platform).

The ATP-adenosine and COX-2/PGE2 pathways constitute critical signaling axes of immunosuppressive metabolites within the TME of gastric cancer. Through a cascade of reactions involving substrate transformation, receptor binding, and signal transduction, these pathways effectively suppress immune function across various dimensions and promote the accumulation of immunosuppressive cells. These cells collaboratively contribute to the establishment of an immunosuppressive network. **Figure 3** illustrates the core mechanisms, immune regulatory effects, and key targeted intervention points of these signaling axes.

### 5.5 The metabolic-immune combined therapy strategy

The integration of targeted metabolic reprogramming with ICIs represents a promising therapeutic approach for gastric cancer. This strategy seeks to augment the efficacy of immunotherapy by modulating tumor metabolic pathways and the immune microenvironment. In recent years, ICIs have achieved significant breakthroughs in the treatment of various cancers. Nevertheless, the effectiveness of ICIs is often constrained by metabolic reprogramming within the TME, a critical factor contributing to the failure of immunotherapy (149, 150).

In gastric cancer, metabolic reprogramming is primarily characterized by an increased reliance on aerobic glycolysis (the Warburg effect). This metabolic shift is recognized as a key driver of tumor progression and immune escape (151, 152). Furthermore, alterations in amino acid, lipid, and nucleotide metabolism also critically contribute to the metabolic reprogramming landscape of gastric cancer (153, 154). Targeting these dysregulated metabolic pathways offers a viable strategy to enhance the efficacy of immune checkpoint blockade. For example, the dual arginase inhibitor OATD-02 restores L-arginine availability in the TME, thereby relieving immunosuppression and potentiating the effect of ICIs (114). Additionally, therapeutic interventions targeting glucose and glutamine metabolism can synergize with PD-1/PD-L1 blockade to augment anti-tumor immune responses (150). Detailed clinical trial data, research progress and translational challenges of above targeted metabolic agents are summarized in the **Supplementary Text 1**.

### 5.6 Clinical application prospects of metabolic markers in the gastric cancer TME

#### 5.6.1 Lactate metabolism-related genes (LMRGs) as predictive biomarkers

Lactate is no longer regarded merely as a metabolic byproduct; rather, it functions as a pivotal signaling molecule that facilitates immunosuppression and tumor invasion. Recent multi-omics analyses have identified lactate metabolism-related gene (LMRG) signatures as robust prognostic and predictive markers (155). Lactic acid metabolism plays a significant role in tumor progression, and the expression patterns of its associated genes offer valuable prognostic and therapeutic response insights for patients with gastric cancer. Firstly, the expression of lactate metabolism-related genes in gastric cancer is intricately linked to the characteristics of the TME. Research indicates that lactic acid metabolism not only influences tumor cell proliferation and migration but also promotes tumor immune evasion by modulating the immune microenvironment. For instance, the expression of lactate metabolism-related genes, such as SLC16A7, can enhance the expression of PD-L1, thereby augmenting the immune evasion capabilities of tumor cells (156). Secondly, LMRGs can serve as independent prognostic indicators in patients with gastric cancer. By analyzing the expression of these genes, researchers have developed various predictive models that effectively stratify patients into high-risk and low-risk groups, enabling predictions regarding survival rates and treatment responses. For instance, models based on lactate metabolism-related genes can accurately predict patients’ responses to immunotherapy, with those in the high-risk group generally exhibiting a poorer prognosis (157, 158). In summary, LMRGs play an important role in the TME of gastric cancer, and their potential as predictive biomarkers provides a new direction for prognosis evaluation and personalized treatment of gastric cancer.

#### 5.6.2 The expression level of IDO1 as a predictor of prognosis and therapeutic response

The expression of IDO1 in gastric cancer is intricately linked to immune evasion within the TME. Research has demonstrated that elevated IDO1 expression is significantly correlated with poor prognosis in patients with gastric cancer, and IDO1 serves as an independent prognostic factor (159). Furthermore, IDO1 expression is co-expressed with PD-1 in tumor-associated macrophages, further revealing the role of IDO1 in facilitating immune escape (160).

IDO1 plays a significant role in the prognosis of gastric cancer and may also influence treatment response. Research indicates that elevated IDO1 expression is associated with increased sensitivity to chemotherapeutic agents, although it may concurrently contribute to resistance against ICIs (160). Furthermore, the positive feedback mechanism involving the MAPK pathway between IDO1 and COL12A1 facilitates gastric cancer metastasis, suggesting that IDO1 could serve as a potential therapeutic target (161). In the context of gastric cancer immunotherapy, the combined assessment of IDO1 and other immune markers may enhance prognostic accuracy. For instance, evaluating IDO1 alongside CD8^+^ T cell levels could serve as a straightforward and effective prognostic biomarker for gastric cancer, as well as predict the efficacy of neoadjuvant chemotherapy (159). Overall, IDO1 expression levels hold significant potential in prognostic evaluation and treatment response prediction for gastric cancer. By integrating IDO1 with other immune markers, novel insights and strategies for the personalized treatment of gastric cancer patients may be developed.

## 6 Prospect and conclusion

This review systematically delineates the cascade network of nutrient competition–metabolic crosstalk–immune escape within the immunosuppressive microenvironment of gastric cancer. It reveals that metabolic reprogramming serves not only as an intrinsic adaptive strategy of tumor cells but also as a core driver that reshapes the immune microenvironment and promotes systemic immunosuppression. The metabolic interplay involving glucose, amino acids, and lipids establishes an ecological barrier in the TME through synergistic signaling driven by substrate depletion and immunosuppressive metabolites (e.g., lactate, adenosine, kynurenine). This understanding offers a metabolic perspective that extends beyond genomic alterations for interpreting primary and secondary resistance to immunotherapy in gastric cancer, thereby laying a theoretical foundation for developing combined “metabolism–immunity” intervention strategies.

Despite advancements, current research continues to encounter several significant challenges. Firstly, the metabolic heterogeneity of the TME in gastric cancer is notably high. Distinct anatomical sites, such as primary and metastatic foci, along with various molecular subtypes, including Epstein-Barr virus (EBV) positive and microsatellite instability-high (MSI-H), exhibit unique metabolic-immune profiles. EBV+ gastric cancer is characterized by a highly immunogenic microenvironment, which is enriched with tumor-infiltrating lymphocytes and exhibits upregulated expression of PD-L1 (162, 163). Recent studies indicate that EBV infection induces specific metabolic and epigenetic changes, including NF-κB/TAP1-mediated immune evasion and DNA demethylation, thereby intricately linking viral metabolism to immune escape mechanisms (164, 165). As a result, patients with EBV+ gastric cancer frequently demonstrate superior clinical responses to ICIs such as nivolumab (166). In contrast, MSI-H gastric cancer is driven by deficiencies in mismatch repair and is characterized by a high tumor mutational burden, which generates numerous neoantigens that demand substantial metabolic resources (163). Despite general susceptibility to ICIs, significant intra-tumoral metabolic heterogeneity and heterogenous mismatch repair status within MSI-H tumors can lead to unpredictable therapeutic responses, necessitating customized metabolic-immune interventions (167).

Furthermore, the metabolic heterogeneity between primary and metastatic gastric cancer further increases the complexity of therapeutic intervention. Primary gastric tumors primarily depend on enhanced glycolysis; however, metastatic cells must adjust dynamically to the nutrient conditions of their secondary environments (168). In the case of peritoneal metastasis, tumor cells encounter a distinct lipid-rich and highly immunosuppressive pre-metastatic niche (169). Within this environment, disseminated gastric cancer cells exploit peritoneal resident macrophages, inducing a metabolic reprogramming that transforms them into a pro-tumor M2-like phenotype, thereby promoting immune evasion (170). Furthermore, in liver metastases, tumor cells also demonstrate metabolic reprogramming. Research indicates that these cells may preferentially utilize fatty acid metabolism over glycolysis in the liver, a metabolic adaptation that supports their survival and proliferation in the new microenvironment (171). The metabolic heterogeneity observed between primary and metastatic lesions suggests that a uniform metabolic targeting strategy is insufficient for effective intervention across all tumor sites. Consequently, it is imperative to develop individualized treatment plans tailored to the distinct metabolic profiles of each lesion. This approach offers crucial theoretical support for enhancing the spatial precision of metabolic immune interventions in the treatment of gastric cancer.

Secondly, the dynamic evolution of metabolic networks, exemplified by treatment-induced metabolic reprogramming and compensatory redundancy, such as the cross-regulation of mTORC1, AMPK, and HIF-1α pathways, constrains the effectiveness of single-target interventions. Furthermore, there is a pronounced bottleneck in clinical translation. The absence of biomarkers for real-time monitoring of the TME’s metabolic state and patient stratification models to predict responses to metabolic interventions impedes the progression from mechanistic research to clinical application.

Future research may be directed towards several key areas: Firstly, a comprehensive analysis of the regulatory networks of core metabolic hubs, such as mTORC1 and HIF-1α, is essential to elucidate the critical nodes involved in metabolic crosstalk between cells. Secondly, the development of multi-target combination strategies and the design of combination therapies based on the synergistic mechanisms of metabolic networks are crucial to overcoming compensatory drug resistance. Thirdly, employing multi-omics technologies to screen for specific metabolic markers could facilitate the stratification and personalization of patient treatment. Fourthly, enhancing the synergistic application of metabolic interventions, ICIs, and cell therapy, while alleviating metabolic restrictions to restore immune cell function, could potentially overcome current therapeutic bottlenecks. As our understanding of the TME metabolic reprogramming network in gastric cancer deepens, the integration of metabolic and immune therapies is anticipated to be a significant breakthrough in improving the prognosis of patients with advanced gastric cancer. This approach provides a scientific foundation and novel clinical insights for enhancing patient outcomes.
