# Supplementary material for: Metabolic reprogramming networks in the gastric cancer tumor microenvironment: an integrated axis of nutrient competition, metabolic crosstalk, and immunosuppression
Source: Front Immunol. 2026 Jul 9;17:1801318. doi: 10.3389/fimmu.2026.1801318 (PMC13391511; doi:10.3389/fimmu.2026.1801318)
Supplement: Supplementary file 1 [file DataSheet1.docx]

**Clinical research progress and translational challenges**

While the aforementioned treatment strategies have demonstrated promising efficacy in preclinical studies, their translation into clinical practice remains at various stages of development, with most still in the early phases. A comprehensive understanding of the current clinical landscape is essential for evaluating the therapeutic potential of metabolic interventions in gastric cancer.

1 IDO1 inhibitors

Epacadostat, a potent and selective oral IDO1 inhibitor, has been extensively evaluated in combination with immune checkpoint inhibitors. The phase I/II ECHO-207/KEYNOTE-723 trial (NCT03085914) assessed epacadostat (100 mg twice daily) combined with pembrolizumab and chemotherapy in 70 patients with advanced solid tumors (1). The regimen demonstrated an acceptable safety profile with an overall response rate (ORR) of 31.4% across all treatment groups. Notably, grade 3–4 treatment-emergent adverse events occurred in 78.6% of patients, with neutropenia being the most common (1). In another phase I/II study (ECHO-206, NCT03308641), the combination of epacadostat with azacitidine and pembrolizumab in 70 heavily pretreated patients with advanced solid tumors yielded an ORR of only 5.7% (four partial responses), indicating limited clinical activity in immunotherapy-experienced populations (2).

2 Arginase inhibitors

A Phase I non-randomized, open-label study (NCT02903914) targeting patients with advanced or metastatic solid tumors evaluated the safety, pharmacokinetics, pharmacodynamics, and efficacy of the arginase 1 (ARG1) inhibitor CB-1158, both as a monotherapy and in combination with pembrolizumab (3). The study cohort comprised 107 patients with locally advanced or metastatic solid tumors. The findings indicated that CB-1158 was generally well tolerated, with the maximum tolerated dose not being reached. The incidence of treatment-related adverse events of grade 3 or higher was 45.8% for monotherapy and 51.7% for combination therapy. Pharmacodynamic analysis demonstrated a dose-dependent increase in plasma arginine levels. Nevertheless, the anti-tumor activity of CB-1158 was limited, with an objective response rate (ORR) of 1.4% for monotherapy, and no significant improvement in therapeutic efficacy was observed when combined with pembrolizumab. In contrast, OATD-02, a first-in-class dual ARG1/ARG2 inhibitor with enhanced cellular penetration, has entered clinical evaluation. The latest preclinical studies demonstrated superior antitumor efficacy compared with ARG1-selective inhibitors, particularly in combination with anti-PD-1 therapy (4). The ongoing phase I/II trial (NCT05759923) aims to evaluate the safety, pharmacokinetics, and preliminary efficacy of OATD-02 in patients with advanced solid tumors, with results anticipated to guide future combination strategies (4).

3 Glutaminase inhibitors

Telaglenastat (CB-839), a glutaminase 1 inhibitor, is currently under investigation in multiple phase I/II clinical trials. One such trial (NCT04310397) examined the combination of CB-839 and nivolumab in a cohort of 118 patients diagnosed with metastatic melanoma, renal cell carcinoma, and non-small cell lung cancer (NSCLC) (5). The combination therapy was generally well-tolerated, with fatigue (42.4%), nausea (39%), and photophobia (32.2%) being the most commonly reported adverse events. Nevertheless, the overall response rate (ORR) was limited to 8.4% across all cohorts, with the most pronounced efficacy observed in checkpoint inhibitor-naïve clear-cell renal cell carcinoma, which exhibited a 24% ORR. Importantly, no responses were recorded in NSCLC patients who had previously undergone anti-PD-1/PD-L1 therapy, highlighting the difficulty in overcoming acquired resistance. Additionally, the combination of CB-839 with capecitabine in colorectal cancer (NCT02861300) was well-tolerated at biologically active doses, with exploratory analyses indicating a potentially enhanced benefit in tumors harboring PIK3CA mutations (6).

4 CD73 inhibitors and CD39 inhibitors

AB680 is a highly potent small molecule inhibitor of CD73, currently undergoing evaluation in phase I/II clinical trials. Preclinical investigations have demonstrated that AB680 effectively inhibits CD73 enzyme activity on human and mouse CD8⁺ T cells, thereby restoring T cell proliferation, cytokine secretion, and cytotoxicity in vitro (7). In a murine melanoma model, administration of AB680 as a monotherapy resulted in delayed tumor growth, while its combination with a PD-1 antibody significantly reduced tumor burden and extended survival (7). Ongoing clinical trials are assessing the efficacy of AB680 in combination with chemotherapy and immune checkpoint inhibitors for the treatment of pancreatic cancer (NCT04104672), metastatic colorectal cancer (NCT04660812), and prostate cancer (NCT04381832). Additionally, another preclinical study indicated that AB598, a CD39 inhibitor, completely inhibits CD39 enzyme activity within tumors, increases intratumoral ATP levels, reduces extracellular CD39 expression on tumor cells, and ultimately controls tumor progression (8). A phase I/1b clinical trial (NCT05891171) is currently being conducted to evaluate the safety and tolerability of AB598, both as a monotherapy and in combination, in patients with advanced malignant tumors.

5 Lactate transport inhibition

A Phase I dose-escalation study was conducted to assess the safety profile of the oral monocarboxylate transporter 1 (MCT1) inhibitor, AZD3965, in patients with advanced solid tumors or lymphoma (9). The study aimed to establish the maximum tolerated dose (MTD) or the recommended Phase II dose (RP2D) and to investigate the impact of inhibiting MCT1-mediated lactate transport on tumor cells. The study cohort comprised 40 patients who received AZD3965 at doses ranging from 5-30 mg once daily or 10-15 mg twice daily. The majority of treatment-related adverse events were of Grade 1 or 2 severity, with common occurrences including electroretinogram changes (retinopathy), fatigue, anorexia, and constipation. The MTD and RP2D were ultimately determined to be 10 mg administered twice daily. Regarding therapeutic efficacy, among the 39 evaluable patients, 9 (23.1%) exhibited the best response, 18 (46.2%) experienced disease progression, 3 (7.7%) had early progression, and 9 patients only underwent baseline disease assessment, precluding evaluation of remission. This study substantiates MCT1 as a viable target for cancer therapy, demonstrating that its inhibitors, such as AZD3965, can disrupt tumor metabolism by obstructing lactate efflux.

6 COX-2 inhibitors

Celecoxib, a selective COX-2 inhibitor, has garnered significant attention in recent years for its potential therapeutic applications in the treatment of cancers. Research indicates that celecoxib may augment the cytotoxic effects of chemotherapeutic agents by suppressing COX-2 expression, particularly in drug-resistant gastric cancer cells (10). Furthermore, celecoxib has been shown to impede tumor angiogenesis by decreasing serum levels of VEGF and COX-2, which may consequently lower the risk of tumor recurrence and metastasis (11).

In a multicenter, randomized phase II trial (12), a cohort of 200 patients diagnosed with metastatic or postoperative recurrent gastric cancer was randomly assigned to either an experimental group, receiving a combination of celecoxib and chemotherapy (n=100), or a control group, receiving chemotherapy alone (n=100). Ultimately, 176 patients completed the treatment protocol, with 89 patients in the experimental group and 87 in the control group. The findings indicated that the median progression-free survival (PFS) for the experimental and control groups was 6 months and 5 months, respectively (*P*=0.73), while the median overall survival (OS) was 12 months and 10 months, respectively (*P*=0.59), with neither result reaching statistical significance. Conversely, within the COX-2 positive subgroup, comprising 52 patients in the experimental group and 50 in the control group, the median PFS in the experimental group was significantly extended to 7.5 months compared to 5 months in the control group (*P*<0.001), and the median OS was significantly extended to 14 months compared to 10 months in the control group (*P*=0.01). Furthermore, the experimental group demonstrated a significantly higher overall remission rate (39% vs. 30%) and disease control rate (66% vs. 54%) than the control group, with all comparisons yielding P-values less than 0.001. Additionally, there was a notable improvement in the quality of life for patients in the experimental group, particularly in terms of pain and fatigue scores. However, no significant difference was observed in the incidence of adverse reactions between the two groups. These findings suggest that the combination of celecoxib and chemotherapy offers enhanced clinical benefits for patients with COX-2 positive advanced gastric cancer.

While celecoxib has received FDA approval for non-oncological indications, including rheumatoid arthritis and osteoarthritis, its clinical application in the treatment of gastric cancer remains experimental. Nonetheless, as a low-toxicity metabolic targeting agent, the novel approach of combining COX-2 inhibitors with immune checkpoint inhibitors offers a promising avenue for research, warranting further validation through clinical trials (13).

7 Potential toxicity, clinical heterogeneity and transformation challenge of metabolic reprogramming targeted drugs

While the pre-clinical outcomes of drugs targeting metabolic reprogramming are promising, the clinical application of these strategies in gastric cancer remains constrained by several significant challenges, notably the potential toxicity, clinical heterogeneity, and difficulties in clinical translation.

First, systemic toxicity presents a significant challenge in cancer treatment, as many targeted metabolic pathways are not exclusive to tumors but are crucial for maintaining normal tissue homeostasis. For instance, glycolysis inhibitors such as 2-deoxyglucose (2-DG) can disrupt cellular energy metabolism by inhibiting the glycolytic pathway, leading to adverse effects such as fatigue and hypoglycemia (14). Furthermore, 2-DG may induce non-targeted metabolic stress through its inhibition of protein glycosylation and the induction of endoplasmic reticulum stress (15). While metabolically targeted therapies hold promise for the treatment of gastric cancer, their associated adverse reactions cannot be overlooked. Future research should focus on mitigating the risk of these adverse effects to optimize treatment strategies for patients with gastric cancer.

Second, the clinical efficacy of metabolic targeting strategies exhibits heterogeneity and can be occasionally disappointing, underscoring the complexity of the tumor metabolic network. A pertinent example is Epacadostat, a selective IDO1 inhibitor, which initially demonstrated significant immunomodulatory potential in preclinical models and early clinical trials, particularly when combined with the PD-1 inhibitor pembrolizumab, suggesting the potential for enhanced anti-tumor activity (16, 17). However, in the pivotal phase III clinical trial Echo-301/Keynote-252, the combination of epacadostat and pembrolizumab did not improve progression-free survival or overall survival rates in patients with unresectable or metastatic melanoma, showing no significant difference compared to pembrolizumab alone (18). This outcome led to the suspension of other ongoing phase III trials and cast doubt on the efficacy of IDO1 inhibitors as immunotherapeutic strategies (19). Research indicates that the failure of epacadostat may be attributed to its impact on the non-enzymatic functions of IDO1 (20).

Although metabolic reprogramming targeted drugs show prospects in preclinical research, they face many challenges in the process of clinical transformation. Firstly, the metabolic heterogeneity inherent in gastric cancer poses a significant challenge. Gastric cancer encompasses a variety of subtypes, complicating the treatment with metabolically targeted drugs. Secondly, the clinical application of metabolic reprogramming is hindered by issues of specificity and safety. Although drugs targeting metabolic pathways exhibit potential in modulating immune cell function, their specificity and safety require further validation (21). Thirdly, the absence of reliable biomarkers limits the application of metabolic reprogramming in cancer treatment, complicating patient selection and the prediction of therapeutic outcomes (22). Lastly, the redundancy within immunosuppressive metabolic networks—such as concurrent lactic acid accumulation, adenosine production, and kynurenine production—suggests that multiple therapeutic interventions may be necessary to address various metabolic nodes. However, this approach significantly increases the complexity of toxicity management and the potential for drug interactions.

**References:**

1. Powderly JD, Klempner SJ, Naing A, Bendell J, Garrido-Laguna I, Catenacci DVT, et al. Epacadostat Plus Pembrolizumab and Chemotherapy for Advanced Solid Tumors: Results from the Phase I/II ECHO-207/KEYNOTE-723 Study. *The oncologist* (2022) 27(11):905-e848. Epub 2022/09/27. doi: 10.1093/oncolo/oyac174. PubMed PMID: 36156099; PubMed Central PMCID: PMCPMC9632315.

2. Luke JJ, Fakih M, Schneider C, Chiorean EG, Bendell J, Kristeleit R, et al. Phase I/II sequencing study of azacitidine, epacadostat, and pembrolizumab in advanced solid tumors. *British journal of cancer* (2023) 128(12):2227-35. Epub 2023/04/23. doi: 10.1038/s41416-023-02267-1. PubMed PMID: 37087488; PubMed Central PMCID: PMCPMC10241827.

3. Naing A, Papadopoulos KP, Pishvaian MJ, Rahma O, Hanna GJ, Garralda E, et al. First-in-human phase 1 study of the arginase inhibitor INCB001158 alone or combined with pembrolizumab in patients with advanced or metastatic solid tumours. *BMJ oncology* (2024) 3(1):e000249. Epub 2025/01/31. doi: 10.1136/bmjonc-2023-000249. PubMed PMID: 39886141; PubMed Central PMCID: PMCPMC11235002.

4. Grzybowski MM, Uçal Y, Muchowicz A, Rejczak T, Kikulska A, Głuchowska KM, et al. Metabolomic reprogramming of the tumor microenvironment by dual arginase inhibitor OATD-02 boosts anticancer immunity. *Scientific reports* (2025) 15(1):18741. Epub 2025/05/29. doi: 10.1038/s41598-025-03446-1. PubMed PMID: 40437024; PubMed Central PMCID: PMCPMC12119792.

5. Gouda MA, Voss MH, Tawbi H, Gordon M, Tykodi SS, Lam ET, et al. A phase I/II study of the safety and efficacy of telaglenastat (CB-839) in combination with nivolumab in patients with metastatic melanoma, renal cell carcinoma, and non-small-cell lung cancer. *ESMO open* (2025) 10(5):104536. Epub 2025/05/14. doi: 10.1016/j.esmoop.2025.104536. PubMed PMID: 40359708; PubMed Central PMCID: PMCPMC12141888.

6. Zhao Y, Feng X, Chen Y, Selfridge JE, Gorityala S, Du Z, et al. 5-Fluorouracil Enhances the Antitumor Activity of the Glutaminase Inhibitor CB-839 against PIK3CA-Mutant Colorectal Cancers. *Cancer research* (2020) 80(21):4815-27. Epub 2020/09/11. doi: 10.1158/0008-5472.Can-20-0600. PubMed PMID: 32907836; PubMed Central PMCID: PMCPMC7642187.

7. Piovesan D, Tan JBL, Becker A, Banuelos J, Narasappa N, DiRenzo D, et al. Targeting CD73 with AB680 (Quemliclustat), a Novel and Potent Small-Molecule CD73 Inhibitor, Restores Immune Functionality and Facilitates Antitumor Immunity. *Molecular cancer therapeutics* (2022) 21(6):948-59. Epub 2022/04/12. doi: 10.1158/1535-7163.Mct-21-0802. PubMed PMID: 35405741; PubMed Central PMCID: PMCPMC9381133.

8. Anderson AE, Parashar K, Jin K, Clor J, Stagnaro CE, Vani U, et al. Characterization of AB598, a CD39 Enzymatic Inhibitory Antibody for the Treatment of Solid Tumors. *Molecular cancer therapeutics* (2024) 23(10):1471-82. Epub 2024/05/27. doi: 10.1158/1535-7163.Mct-23-0865. PubMed PMID: 38797955; PubMed Central PMCID: PMCPMC11443198.

9. Halford S, Veal GJ, Wedge SR, Payne GS, Bacon CM, Sloan P, et al. A Phase I Dose-escalation Study of AZD3965, an Oral Monocarboxylate Transporter 1 Inhibitor, in Patients with Advanced Cancer. *Clinical cancer research : an official journal of the American Association for Cancer Research* (2023) 29(8):1429-39. Epub 2023/01/19. doi: 10.1158/1078-0432.Ccr-22-2263. PubMed PMID: 36652553; PubMed Central PMCID: PMCPMC7614436.

10. Xu HB, Shen FM, Lv QZ. Celecoxib enhanced the cytotoxic effect of cisplatin in drug-resistant human gastric cancer cells by inhibition of cyclooxygenase-2. *European journal of pharmacology* (2015) 769:1-7. Epub 2015/09/27. doi: 10.1016/j.ejphar.2015.09.025. PubMed PMID: 26407653.

11. Han X, Li H, Su L, Zhu W, Xu W, Li K, et al. Effect of celecoxib plus standard chemotherapy on serum levels of vascular endothelial growth factor and cyclooxygenase-2 in patients with gastric cancer. *Biomedical reports* (2014) 2(2):183-7. Epub 2014/03/22. doi: 10.3892/br.2013.209. PubMed PMID: 24649093; PubMed Central PMCID: PMCPMC3917754.

12. Guo Q, Li Q, Wang J, Liu M, Wang Y, Chen Z, et al. A comprehensive evaluation of clinical efficacy and safety of celecoxib in combination with chemotherapy in metastatic or postoperative recurrent gastric cancer patients: A preliminary, three-center, clinical trial study. *Medicine* (2019) 98(27):e16234. Epub 2019/07/07. doi: 10.1097/md.0000000000016234. PubMed PMID: 31277138; PubMed Central PMCID: PMCPMC6635161.

13. Chen JS, Chou CH, Wu YH, Yang MH, Chu SH, Chao YS, et al. CC-01 (chidamide plus celecoxib) modifies the tumor immune microenvironment and reduces tumor progression combined with immune checkpoint inhibitor. *Scientific reports* (2022) 12(1):1100. Epub 2022/01/22. doi: 10.1038/s41598-022-05055-8. PubMed PMID: 35058524; PubMed Central PMCID: PMCPMC8776878.

14. Laussel C, Léon S. Cellular toxicity of the metabolic inhibitor 2-deoxyglucose and associated resistance mechanisms. *Biochemical pharmacology* (2020) 182:114213. Epub 2020/09/06. doi: 10.1016/j.bcp.2020.114213. PubMed PMID: 32890467.

15. Hong SY, Hagen T. 2-Deoxyglucose induces the expression of thioredoxin interacting protein (TXNIP) by increasing O-GlcNAcylation - Implications for targeting the Warburg effect in cancer cells. *Biochemical and biophysical research communications* (2015) 465(4):838-44. Epub 2015/09/01. doi: 10.1016/j.bbrc.2015.08.097. PubMed PMID: 26315267.

16. Yue EW, Sparks R, Polam P, Modi D, Douty B, Wayland B, et al. INCB24360 (Epacadostat), a Highly Potent and Selective Indoleamine-2,3-dioxygenase 1 (IDO1) Inhibitor for Immuno-oncology. *ACS medicinal chemistry letters* (2017) 8(5):486-91. Epub 2017/05/20. doi: 10.1021/acsmedchemlett.6b00391. PubMed PMID: 28523098; PubMed Central PMCID: PMCPMC5430407.

17. Beatty GL, O'Dwyer PJ, Clark J, Shi JG, Bowman KJ, Scherle PA, et al. First-in-Human Phase I Study of the Oral Inhibitor of Indoleamine 2,3-Dioxygenase-1 Epacadostat (INCB024360) in Patients with Advanced Solid Malignancies. *Clinical cancer research : an official journal of the American Association for Cancer Research* (2017) 23(13):3269-76. Epub 2017/01/06. doi: 10.1158/1078-0432.Ccr-16-2272. PubMed PMID: 28053021; PubMed Central PMCID: PMCPMC5496788.

18. Long GV, Dummer R, Hamid O, Gajewski TF, Caglevic C, Dalle S, et al. Epacadostat plus pembrolizumab versus placebo plus pembrolizumab in patients with unresectable or metastatic melanoma (ECHO-301/KEYNOTE-252): a phase 3, randomised, double-blind study. *The Lancet Oncology* (2019) 20(8):1083-97. Epub 2019/06/22. doi: 10.1016/s1470-2045(19)30274-8. PubMed PMID: 31221619.

19. Komiya T, Huang CH. Updates in the Clinical Development of Epacadostat and Other Indoleamine 2,3-Dioxygenase 1 Inhibitors (IDO1) for Human Cancers. *Frontiers in oncology* (2018) 8:423. Epub 2018/10/20. doi: 10.3389/fonc.2018.00423. PubMed PMID: 30338242; PubMed Central PMCID: PMCPMC6180183.

20. Panfili E, Mondanelli G, Orabona C, Gargaro M, Volpi C, Belladonna ML, et al. The catalytic inhibitor epacadostat can affect the non-enzymatic function of IDO1. *Frontiers in immunology* (2023) 14:1134551. Epub 2023/05/01. doi: 10.3389/fimmu.2023.1134551. PubMed PMID: 37122718; PubMed Central PMCID: PMCPMC10145169.

21. Hu Y, Zhao Q, Dai H, Wu Y, Tang X, Zhang N, et al. Metabolic reprogramming as a therapeutic target for modulating the Th17/Treg balance in autoimmune diseases: a comprehensive review. *Frontiers in immunology* (2025) 16:1687755. Epub 2026/01/01. doi: 10.3389/fimmu.2025.1687755. PubMed PMID: 41476956; PubMed Central PMCID: PMCPMC12747992.

22. Hong M, Baek JH. Targeting AMPK for Cancer Therapy: Metabolic Reprogramming as a Therapeutic Strategy. *Oncology research* (2025) 33(10):2699-724. Epub 2025/10/06. doi: 10.32604/or.2025.067487. PubMed PMID: 41050082; PubMed Central PMCID: PMCPMC12494096.
